# *Capnocytophaga ochracea* Septicemia After a Dog Bite: The Case of a Usual Suspect Transmitting an Unusual Organism

**DOI:** 10.5811/cpcem.5826

**Published:** 2024-07-11

**Authors:** Clifford Chang, Vakula Atthota, Madison Lord, Michael P. Bonk, Muhammad Durrani

**Affiliations:** * Inspira Health Network, Department of Emergency Medicine, Vineland, New Jersey; † Inspira Health Network, Department of Infectious Diseases, Vineland, New Jersey; ‡ Inspira Health Network, Department of Internal Medicine, Vineland, New Jersey; § Rowan University, Cooper Medical School, Division of Critical Care Medicine, Camden, New Jersey

**Keywords:** *capnocytophaga*, *antibiotic resistance*, *dog bite*, *purpura fulminans*, *immunocompromise*, extended-spectrum β-lactamases, *ESBL*

## Abstract

**Introduction:**

*Capnocytophaga ochracea* is found in the human oral microbiome and is a rare cause of antibiotic-resistant, opportunistic septicemia in immunocompromised hosts. The zoonotic transmission of *C ochracea* from canines to humans has not yet been reported in the literature. Cohabitation with people is associated with oral colonization in dogs and may be a reservoir for *Capnocytophaga* infections, which have a decreased susceptibility to first-line antibiotics commonly used to treat animal exposures.

**Case Report:**

This is the case of a 70-year-old male with a remote history of lymphoma status post splenectomy, in remission, who presented with stigmata of *Capnocytophaga* septicemia after a dog bite, which included purpura fulminans on physical examination. Initial broad-spectrum coverage with cefepime failed to slow the progression into multiorgan failure. A *Capnocytophaga* strain with extended resistance was suspected. Antibiotics were transitioned to meropenem, and the patient eventually made a good recovery. Blood cultures isolated *C ochracea.*

**Conclusion:**

*Capnocytophaga* infections should be suspected in patients with severe sepsis and purpura fulminans after a canine exposure. Canine pets may be a reservoir for *Capnocytophaga* species with increased antibiotic resistances, such as *C ochracea*, which trace their origins to the human oral microbiome. A thorough medical history is essential to identify risk factors such as asplenia and active immune compromise that are associated with infections from antibiotic-resistant strains and worse outcomes. For *Capnocytophag*a infections that fail initial therapies, cephalosporins should be avoided because of high resistance rates, and the use of carbapenems may be favored over combination beta-lactam/beta-lactamase inhibitors in select clinical scenarios.

Population Health Research Capsule What do we already know about this clinical entity?

*Human oral bacteria are often resistant to conventional antibiotics. Cohabitation is associated with the oral colonization of these bacteria in dogs.*

What makes this presentation of disease reportable?

*This is the first reported case of a human oral Capnocytophaga species causing sepsis after zoonotic transmission via a dog bite.*

What is the major learning point?

*Pet bites may transmit antibiotic-resistant bacteria that originate from the human oral microbiome. The use of carbapenems should be considered when initial therapies fail.*

How might this improve emergency medicine practice?

*Clinicians should recognize the changing nature of infectious diseases and understand the role of extended spectrum antimicrobials.*

## INTRODUCTION

*Capnocytophag*a is a genus of encapsulated, Gram-negative rods that resides in the commensal mammalian flora as facultative anaerobes. Native to the canine oral microbiome, *C canimorsus* is well known to cause infections in humans through canine exposures such as bites, licks, and scratches. Other species of *Capnocytophaga* such as *C ochracea, C sputigena, and C gingivalis* are native to the human oral microbiome and often found in association with gingivitis.^1^ Bacteremia from these human oral *Capnocytophaga* (HOC) species disproportionally affects severely immunocompromised patients and is associated with poor outcomes. A retrospective study published in 2021 by Chesdachai et al found that the six-month mortality of patients with HOC bacteremia was higher than those with *C canimorsus* bacteremia, 36.4% vs 6.2%, respectively.^2^

Potentiated by a compromised immune system, the pathogenesis of HOC bacteremia likely involves self-seeding from the oral cavity into the systemic circulation. While the majority of *C canimorsus* infections are associated with known animal exposures (up to 77%), the zoonotic transmission of HOC species such as *C ochracea* has not yet been reported in the literature.^2^ Interestingly, *C ochracea* along with other human oral microbes are found in the mouths of dogs that cohabitate with people.^3^ ^,^ ^4^ Such pets may be a reservoir of potentially pathogenic bacteria that do not yet have a track record for causing zoonotic infections.

The poor outcomes associated with *C ochracea* and other HOC infections are multifactorial and include the degree of host immune suppression, the presence of underlying diseases such as hematologic malignancy, and increased antibiotic resistance. Unlike *C canimorsus*, which is almost universally susceptible to narrow-spectrum antibiotics (eg, penicillin), *C ochracea* and other HOC species are often resistant to commonly used broad-spectrum antibiotics for polymicrobial animal exposures. Up to 70% of HOC isolates produce beta-lactamases, which confer resistance to first-generation cephalosporins (100% resistant), amoxicillin (86%), and third-generation cephalosporins (63%).^5^ ^–^ ^7^

Although *Capnocytophaga* species are typically sensitive to combination beta-lactam/beta-lactamase inhibitors in vitro, treating critically ill, bacteremic patients with antibiotics such as amoxicillin-clavulanate or piperacillin-tazobactam may result in poor outcomes. A noninferiority trial published in 2019 randomized 378 patients with Gram-negative bacteremia demonstrating extended spectrum beta-lactamase activity (ESBL), defined as resistance to ceftriaxone, to either treatment with piperacillin-tazobactam or meropenem. Despite having confirmed in vitro susceptibly to both antibiotics, patients treated with piperacillin-tazobactam (12.3%) had increased 30-day mortality when compared to meropenem (3.7%).^8^

Herein we describe the first reported case to our knowledge of human oral-associated *Capnocytophaga* bacteremia transmitted from an animal. The patient described in this case developed severe *C ochracea* septicemia with purpura fulminans after a dog bite and eventually had a good outcome after treatment with meropenem.

## CASE REPORT

**Image 1.**
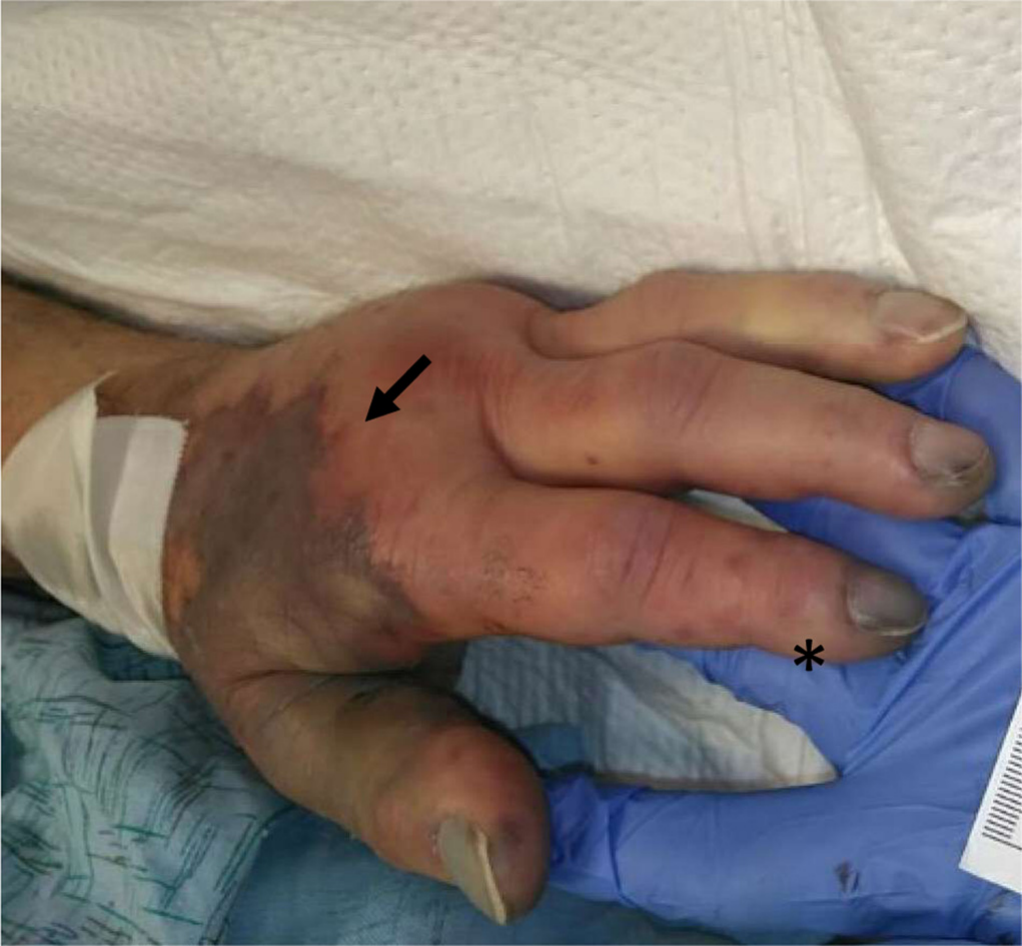
Left hand with purpura and local necrosis (arrows) as well as petechiae (asterisk).

**Image 2.**
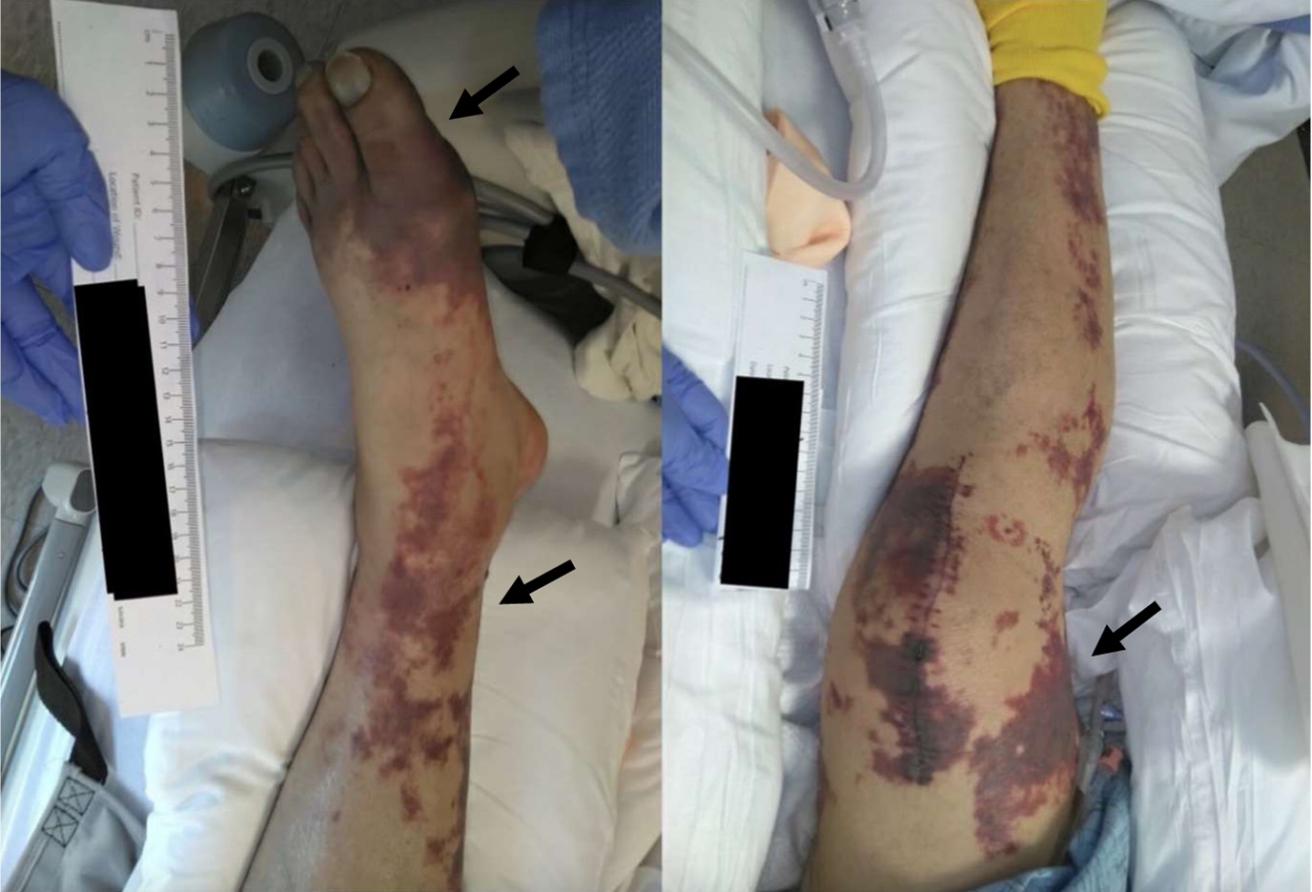
Pupura fulminans (arrows) of the left and right lower extremities, respectively.

A 70-year-old male with a history of Stage III diffuse large B-cell lymphoma status post chemotherapy and splenectomy 20 years prior, currently in remission, presented to a large community emergency department with generalized weakness and altered mental status three days after he sustained a bite to his left thumb from a pet dog. The dog was fully vaccinated and had not been demonstrating abnormal behaviors leading up to the incident. On arrival the patient appeared acutely ill. Vitals signs were notable for a heart rate of 108 beats per minute, blood pressure of 78/43 millimeters of mercury, tachypnea at a rate of 38 breaths per minute, and a temperature of 36.3° Celsius. Examination of the ulnar aspect of the left thumb revealed two faint, punctate bite marks that later became dusky and locally necrotic (Image 1). Dark purple, non-blanching macules were seen in all extremities consistent with purpura fulminans and highly suggestive of *Capnocytophaga* septicemia (Image 2).

The initial lactic acid was 15.0 millimoles per liter (mmol/L) (reference range: 0.5–1.5 mmol/L), and the white blood cell count reached a peak of 90.0 × 10^9^ cells/L (4.5 – 11.0 × 10^9^ cells/L). Given the severity of the patient’s illness, vancomycin and cefepime were initiated empirically. Despite broad spectrum antibiotics and the resuscitation of septic shock with crystalloid fluids, glucocorticoid therapy, and escalating vasoactive medications, the patient deteriorated into multiorgan failure. Within the first day of admission the patient was intubated, placed on mechanical ventilation, initiated on renal replacement therapy, developed coagulopathy, and required intravenous inotropic support. Echocardiogram demonstrated severe global hypokinesis of the left ventricle with a newly depressed ejection fraction of 20–25% consistent with acute septic cardiomyopathy.

Encapsulated *Capnocytophaga* infection remained highest on the differential given the patient’s history of asplenia, recent canine exposure, and purpura fulminans on physical examination. However, the lack of response to cefepime raised concerns for the presence of a resistant *Capnocytophaga* species, potentially with extended spectrum activity. The patient’s treatment regimen was transitioned to meropenem one gram every eight hours on which he began to demonstrate clinical improvement.

*Capnocytophaga* are fastidious, slow-growing bacteria, and our suspicion for this organism was communicated to the microbiology laboratory to increase culture yields. Additional growth media were used, and the specimens were observed for a longer duration. Gram-negative bacilli were found in the aerobic bottles after five days, and *C ochracea* finally speciated after 11 days. The speciation was confirmed by both biochemical methods, using RapID ANA II (Thermo Fisher Scientific Inc, Waltham, MA) and mass spectrometry matrix-assisted laser desorption/ionization (Bruker Corporation, Billerica, MA). Susceptibilities studies were unfortunately not performed given difficulty of culturing the organism.

After a gradual recovery, the patient was transferred out of the intensive care unit nine days after admission and discharged back home on day 18 with visiting rehabilitation services as well as the remainder of a four-week total course of meropenem. At discharge, he was ambulatory and had a recovered left ventricular ejection fraction of 50–55%. Other sequelae included multiple toe amputations for vasopressor and coagulopathy-associated gangrene as well as post-debridement contractures of the left hand. Despite his critical illness and prolonged hospitalization, the patient made a remarkable recovery. His goal was to eventually return to work full time.

## DISCUSSION

Although more commonly thought of as a commensal organism in the oral microbiota, HOC species such as *C ochracea* are a rare cause of severe, opportunistic bacteremia in patients with risk factors for active immune suppression. A case series published in 2001 reported that of 28 cancer patients with neutropenic fevers related to HOC bacteremia, 25 (89%) had an active hematologic malignancy and half had moderate to severe mucositis.^9^ Identified in one third of cases, *C ochracea* was the most commonly isolated species. A more recent review published in 2021 found that all 22 patients with HOC bacteremia had at least one risk factor for active immunocompromise, most commonly immunosuppressive medications (72%), hematopoietic stem cell transplantation (54%), and hematologic malignancy (40%).^2^

The patient described in this case underwent chemotherapy and splenectomy for a lymphoma 20 years prior and had been in remission since. Without signs of an active malignancy, his primary risk factor for *Capnocytophaga* sepsis was likely asplenia. In terms of infectious source, the patient exhibited no signs of mucositis and was bitten only three days prior to presentation, which is within the one to seven day incubation period of *Capnocytophaga* infections.^10^

The speciation of *C ochracea* instead of *C canimorsus* from the dog bite was unanticipated. In a retrospective review of *Capnocytophaga* bacteremia, animal exposure was confirmed in 68.8% of *C canimorsus* infections while no patients with infections from other *Capnocytophaga* species had reported exposures.^2^ *Capnocytophaga ochracea* is not thought to be native to the canine oral microbiome; however, several HOC species have been identified in the oral microbiomes of pets.^3^ Cohabitation and close contact may facilitate the transmission of microorganisms from humans to pet animals. Similar to how the human oral and gut microbiome is often transmitted from mothers to their children, there may be transmission of human oral microbes to pet dogs and cats.^4^ ^,^ ^11^ ^,^ ^12^ The potential of pets as reservoirs for HOC species, timing of symptoms following inoculation, and absence of strong risk factors for spontaneous opportunistic bacteremia make the case for zoonosis as the source of our patient’s *C ochracea* sepsis.

The antimicrobial susceptibility of *C ochracea* and other HOC infections differs from that of *C canimorsus. Capnocytophaga canimorsus* strains rarely produce beta-lactamases and are largely susceptible to common antibiotics used in the management of polymicrobial animal exposures (ie, amoxicillin-clavulanate, clindamycin, third-generation cephalosporins). Up to 70% of HOC isolates produce beta-lactamases, and more than 60% of those are resistant to third-generation cephalosporins.^7^ Additionally, there are reports of multidrug resistant strains of *C ochracea, C sputigena*, and *C gingivalis* that are resistant to fourth-generation cephalosporins (eg, cefepime).^13^ ^,^ ^14^ These findings are concerning for extended-spectrum β-lactamases (ESBL) activity among HOC species.

Combination antibiotics (ie, amoxicillin-clavulanate or piperacillin-tazobactam) may appear to be reasonable alternatives based on in vitro susceptibilities; however, the treatment of ESBL Gram-negative bacteremia with piperacillin-tazobactam rather than meropenem is associated with decreased survival.^8^ Increased bacterial density within infected tissue can alter local pharmacokinetics through the well-described “inoculum effect” where sensitivity to piperacillin-tazobactam can decrease from 95% at in vitro bacterial concentrations to 58% at in vivo concentrations.^15^ This effect is limited in carbapenems and appears to be mediated by the downregulation of target proteins, synergistic enzymatic degradation, and biofilm production.

A limitation of our case is the lack of susceptibility data; however, the prevalence of later-generation cephalosporin resistance among *C ochracea* strains and the patient’s lack of clinical improvement with cefepime raised our suspicion for the presence of extended spectrum resistances. In our case, treatment with meropenem ultimately led to a good outcome.

## CONCLUSION

*Capnocytophaga* bacteremia should be suspected in patients with severe sepsis and purpura fulminans after a canine exposure. Resistant infections should be considered in patients who do not respond to initial therapies. Patients with asplenia and active immunocompromise are at highest risk. In such cases, cephalosporins should be avoided because of high resistance rates, and the use of carbapenems may be favored over combination beta-lactam/beta-lactamase inhibitors in select clinical scenarios. Canine pets may be a reservoir for *Capnocytophaga* species with increased resistances, such as *C ochracea*, which trace their origins to the human oral microbiome.
